# Effect of F11R Gene Knockdown on Malignant Biological Behaviors of Pancreatic Cancer Cells

**DOI:** 10.1155/2022/3379027

**Published:** 2022-03-07

**Authors:** HaiDi Zhang, RenDan Zhang, Jiaxin Yao, XianHua Hu, Yu Pu, Shuai He, Jinchuan Yu, Huiling Zhu, Bo Mu, ChunYan Zhao

**Affiliations:** ^1^Basic Medical College, North Sichuan Medical College, 234# Fujiang Road, Shunqing District, Nanchong, Sichuan 637007, China; ^2^Sichuan Key Laboratory of Medical Imaging, Institute of Medical Imaging, North Sichuan Medical College, 234# Fujiang Road, Shunqing District, Nanchong, Sichuan 637007, China; ^3^Department of Clinical Medicine, North Sichuan Medical College, 234# Fujiang Road, Shunqing District, Nanchong, Sichuan 637007, China

## Abstract

F11R receptor (F11R/junctional adhesion molecule-A/F11R-A) is preferentially concentrated at tight junctions and influences epithelial cell morphology and migration. Numerous studies have shown that the aberrant expression of F11R contributes to tumor progression including pancreatic cancer. However, the significance of F11R in various tumors is controversial, and the role of F11R in regulating the malignant behaviors of human pancreatic cancer is unknown. To investigate the role of F11R in the carcinogenesis of pancreatic cancer and the potential targets of F11R as a therapeutic target for pancreatic cancer, we knocked down F11R in the pancreatic cancer cell line PANC-1 using lentiviral approaches. We found that F11R silencing led to decreased cell proliferation, a loss of cell invasiveness, cell cycle arrest in the G1 phase, and enhanced cell apoptosis. The present results suggest that F11R may be a promising therapeutic target for pancreatic cancer.

## 1. Introduction

Pancreatic cancer is one of the most aggressive malignant tumors that remains difficult to diagnose and therapy. Although progress has been made regarding pancreatic cancer epidemiology, the mortality rates of PDAC patients remain high, and the 5-year survival rates are low [1]. Poor prognosis means finding more accurate biomarkers to predict a patient's prognosis and finding a more effective therapeutic gene targets are top priority for current research. Although many studies have proved some carcinogenic mechanisms and molecular mechanisms of pancreatic cancer including genomic instability [2], gene mutation [3], tumor suppressor gene expression, and signaling pathways [4, 5], the overall and exact molecular pathology of pancreatic cancer remains unclear. So, a large number of basic scientific research and studies are still needed.

The human F11R receptor belongs to the immunoglobulin superfamily and promotes tight junction formation between epithelial and endothelial cells [6]. F11R is a 32–35 kDa protein that is widely expressed in neutrophils, monocytes, platelets, and lymphocytes [7]. F11R regulates epithelial and endothelial cell movement, leukocyte migration, platelet activation, and cell barrier integrity. The regulation of F11R is closely related to its ability to dimerize mediated through the intracellular PDZ-binding motif [8]. Recently, F11R has been investigated in various tumors [9–11], but its role in tumorigenesis remains controversial [12, 13]. In renal cell cancer, downregulation of F11R was associated with a more aggressive phenotype [14], whereas there are conflicting data in the literature as to the effects of F11R expression in breast cancer. It is necessary to explore in detail the clinical significance and biological roles of F11R in carcinogenesis and tumor progression. This suggests that the F11R gene may play a role as oncogene in pancreatic cancer cell lines. Therefore, we further explored the effects of the F11R gene on the malignant biological behavior of pancreatic cancer cells by knocking down the F11R gene and establishing a negative control group, laying a foundation for future molecular pathway research.

## 2. Materials and Methods

The pancreatic cancer cell line PANC-1 was purchased from the cell bank of the Chinese Academy of Sciences in Shanghai. Cell lines were cultured in DMEM medium, supplemented with 10% fetal bovine serum FBS (Gibco®, Shanghai, China), 1% penicillin, and 1% streptomycin at 37°C in a 5% CO_2_ incubator.

UALCAN analysis: the expression levels of F11R and tumor grade were analyzed in UALCAN database (https://ualcan.path.uab.edu/) [15]. GEPIA (Gene Expression Profiling Interactive Analysis) (https://gepia.cancer-pku.cn/) [16] is an interactive web application for gene expression analysis based on 9736 tumors and 8587 normal samples from the TCGA (The Cancer Genome Atlas) and the GTEx (Genotype-Tissue Expression) databases. The GEPIA database was used to compare mRNA levels of F11R between tumor and normal tissue in TCGA databases.

To assess the prognostic value of F11R, Kaplan–Meier Plotter (KM plotter) database (KM plotter, https://kmplot.com/analysis/index.php?p=service) [17] was used to explore the association between F11R expression and pancreatic cancer patient outcome.

### 2.1. RNAi Lentivirus Transduction

The complementary DNA sequence (5′-CCGGTTCTCCGAACGTGTCACGTTTCAAGAGAACGTGACACGTTCGGAGAATTTTTG-3′) of F11R was designed from the full-length F11R sequence (GeneID: 50848, NM_016946.6) by GeneChem Co. Ltd. (Shanghai, China). After testing knockdown efficiencies, the stem-loop oligonucleotides were synthesized and inserted into the lentivirus-based PSCSI-GFP (GeneChem Co. Ltd.) with BamH I/EcoRI sites.

Lentivirus infection was performed as per the method described in [18]. PANC1 cells were cultured into 6-well plates and then the F11R-shRNA-lentivirus or negative control (vector) lentivirus was added according to the multiplicity of infection (MOI). After 72 h of infection, the cells were observed under a fluorescence microscope (Olympus, Tokyo, Japan). After 120 h of infection, the cells were harvested to determine knockdown efficiency by quantitative RT-PCR.

### 2.2. Western Blot Analysis

Western blot analysis was performed according to the method described in [19], with minor modifications. Briefly, proteins from infected cells were extracted and protein expression was determined by western blot analysis. Cell lysates containing 5× SDS sample buffer were boiled at 95°C for 5 min, and 30 *μ*g protein was resolved by SDS-PAGE. Proteins were wet-transferred onto a polyvinylidene difluoride membranes (PVDF) at 200 mA for 2 h and blocked in 5% milk in PBS-T for 1 hour. Membranes were probed with rabbit anti-F11R (Co.# 381191, 1 : 1000; Zen BioScience, China) and rabbit anti-GAPDH (Co.#R24404; Zen BioScience, China) overnight at 4°C and labeled with the appropriate secondary antibodies (Co.#511203, 1 : 5000; Zen BioScience, China) at room temperature for 1 h. Protein bands were visualized using the BeyoECL system and imaged on a fluorescence imager.

### 2.3. Cell Proliferation Assays

Cell proliferation was analyzed by Cell Counter Kit-8 (Shanghai Beyotime Biotechnology, China) according to the method described in [20]. Briefly, PANC-1-negative control RNAi-GFP (NC) and stable RNAi-F11R cells were plated at a density of 3 × 10^3^ cells per well of a 96-well plate, and 10 *μ*l of CCK8 reagent was added to the cells for 2 h. Absorbance was measured at 450 nm.

### 2.4. Cell Cycle and Apoptosis Analysis

Cell cycle and apoptosis assays were performed according to the method described in [21]. In brief, cells in the logarithmic phase were detached with trypsin and cell suspensions were treated with Hoechst33342/PI kits (KGA212; Jiangsu KeyGEN BioTECH Corp., Ltd, China). Stained cells were analyzed on ACEA NovoCyte.

### 2.5. Invasion Assays

For transwell assays, refer to the method described in [22]. Briefly, CHEMICON cell invasion assays were performed in an invasion chamber, consisting of a 24-well tissue culture plate with 12 cell culture inserts. The inserts contain an 8 *μ*m pore size polycarbonate membrane, over which a thin layer of ECMatrixTM was dried. The ECM layer occludes the membrane pores, blocking noninvasive cells from migrating through the membrane. Invasive cells can, however, migrate through the ECM layer and cling to the bottom of the polycarbonate membrane. Cells (1 × 10^4^) were added to the transwells, and FBS-containing medium (500 *μ*l) was added to the lower chamber. Cells were cultured for 24 hours, and the upper chamber was stained with 0.1% crystal violet. Migrating cells were imaged on a Leica DC 300F microscope and counted by Image J.

### 2.6. Statistical Analysis

Images were analyzed with Image J (National Institutes of Health). Statistical analysis were performed using SPSS 13.0. Data were compared using an unpaired two-tailed Student's *T*-test. *P* value <0.05 (*∗*) and *P* value <0.01 (*∗∗*) were considered statistically significant.

## 3. Results

### 3.1. Expression of F11R in Pancreatic Cancer

**Figure 1:**
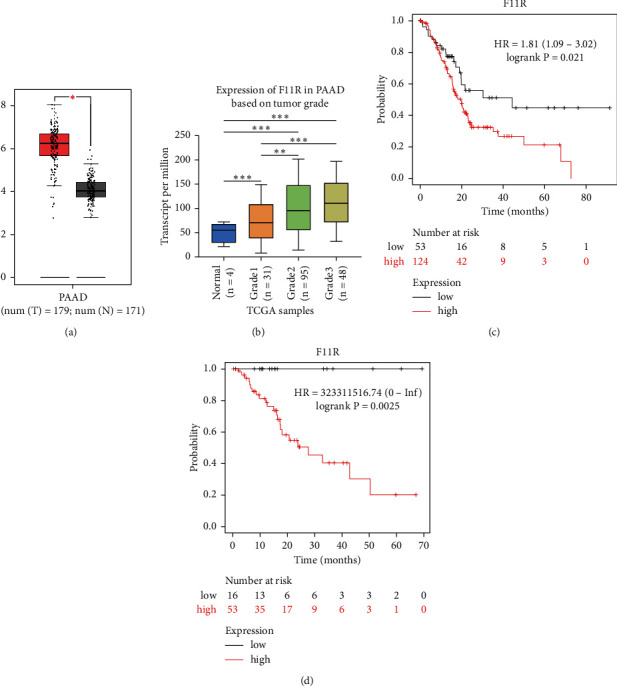
Bioinformatics analysis of F11R in pancreatic cancer. (a) Bioinformatics analysis showed that the expression of F11R in pancreatic cancer was higher than that of normal tissue (*P* < 0.05) using GEPIA from the TCGA database. (b) According to the degree of malignancy, we classified the 174 cases of pancreatic tumors into three grades (grade 1, grade 2, and grade 3). The expression of F11R in pancreatic cancer tissue was significant correlation with tumor grade. (c, d) Kaplan–Meier survival curves of overall survival (OS) and recurrence-free survival (RFS) for high and low F11R expression in pancreatic cancer based on the TCGA database (^*∗*^*P* value <0.05, ^*∗∗*^*P* value <0.01, and ^*∗∗∗*^*P* value <0.001).

Upon GEPIA (https://gepia.cancer-pku.cn/) analysis of F11R expression in 179 pancreatic cancer tissues and 171 normal tissues from TCGA database, the expression of F11R was significantly higher in pancreatic cancer patients (Figure 1(a)). UALCAN (https://ualcan.path.uab.edu/cgi-bin/TCGAExResultNew2.pl?genenam=F11R&ctype=PAAD) analysis showed that F11R expression in the pancreatic cancer tissue also varied according to tumor grade (Figure 1(b)). Furthermore, we found reduced F11R expression to be correlated with poorer patient prognosis in PDAC about OS (HR = 1.81 (1.09–3.02), *P*=0.021) and RFS (HR = 323311516.74 (0-inf), *P*=0.0025) (Figures 1(c)–1(d)). Therefore, we speculate that the expression of F11R is related to the malignant biological behaviors of pancreatic cancer.

### 3.2. Knockdown Efficiency Determined by Cell Fluorescent and Western Blot Analysis

**Figure 2:**
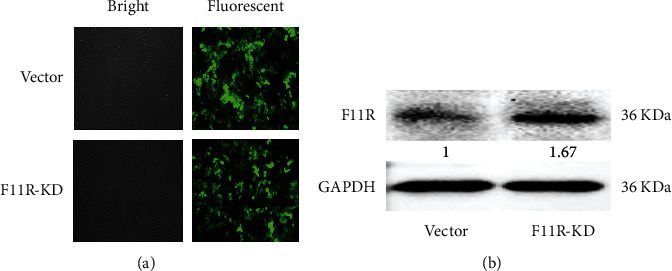
Knockdown of PANC-1 F11R following lentivirus transfection. (a) PANC-1 cells were transfected with control vector and F11R-KD lentiviruses for 72 h GFP fluorescence showed an infection efficiency ≥90%. (b) PANC-1 cells infected with the indicated lentiviruses for 72 h were assessed for F11R expression by western blot analysis. The gray scale of the brane is quantified by IMAGEJ software. F11R expression in the F11R-KD group was significantly reduced.

In order to explore the role of F11R in pancreatic cancer, we knockdown the F11R gene of the PANC-1 cell line with F11R-shRNA lentivirus. NC lentivirus was used as the negative control. As shown in Figure 2(a), infection after three days, the proportion of infected cells was >90%. Western blot analysis showed that the expression of F11R proteins was significantly lower after lentivirus transfection (Figure 2(b)).

### 3.3. Knockdown of F11R Inhibited PANC-1 Cell Proliferation

**Figure 3:**
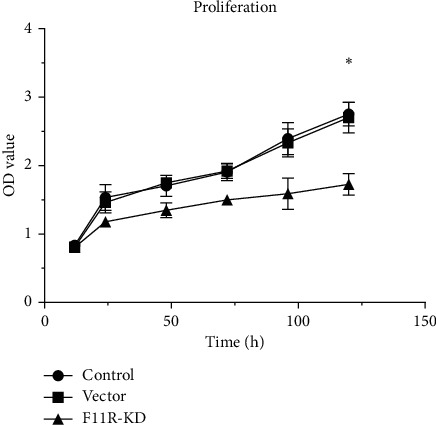
Cell proliferation in the F11R-KD group was significantly slower than in the blank control and vector groups (^*∗*^*P* value <0.05).

Enhanced cell proliferation is a characteristic feature of tumor cells. Cell Counting Kit-8 (Figure 3) was performed to assess the proliferation of PANC-1 with F11R knockdown cells. Comparable proliferation rates were observed between control and vector cells. Cell numbers in the F11R knockdown group were significantly lower than the control and vector groups, indicating reduced proliferation (*P* < 0.05).

### 3.4. Knockdown of F11R Affected PANC-1 Cell Cycle Progression

**Figure 4:**
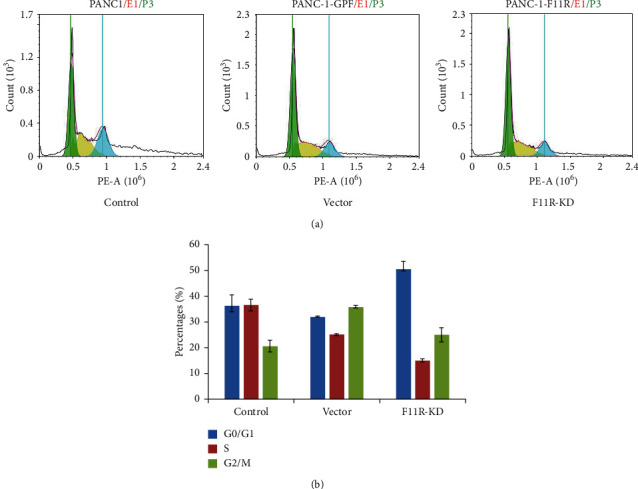
Flow cytometry analysis. Transfection increased the number of cells in the G0/G1 phase. Cytotoxicity was significantly higher in F11R-KD cells compared to control or vector groups. F11R silencing led to further G0/G1 phase arrest (^*∗*^*P* value <0.05).

Flow cytometry (FCM) was used to assess the role of F11R in cell cycle of PANC-1 cells. As shown in Figures 4(a) and 4(b), the F11R knockdown cells significantly blocked pancreatic cancer cell cycle at G0/G1 phase (control group 36.29 ± 4.11%, vector group 31.98 ± 0.16%, and F11R-KD group 50.56 ± 2.96%), with statistically significant differences (*P* < 0.05). At the same time, G2 cells were significantly reduced (control group 36.42 ± 2.29%, vector group 25.01 ± 0.32%, and F11R-KD group 15.08 ± 0.6%), while S cells had no significant effect (control group 20.47 ± 2.25%, vector group 35.85 ± 0.38%, and F11R-KD group 29.78 ± 2.8%), and there was no statistical difference (*P* > 0.05).

### 3.5. Knockdown of F11R Induced PANC-1 Cell Apoptosis

**Figure 5:**
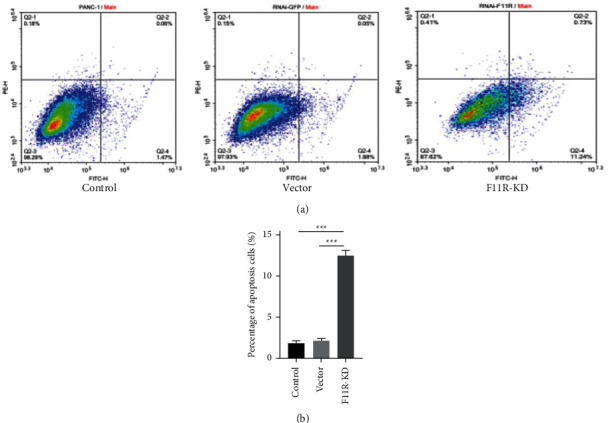
Apoptosis assessments. The number of apoptotic cells significantly increased following F11R silencing (lower right quadrant), as did the number of necrotic cells (upper right quadrant). Compared with the control group and vector group, the proportion of dead cells in F11R-KD cells increased, with statistically significant differences (^*∗∗∗*^*P* value <0.001).

Cancer cells are typically refractory to apoptotic stimuli. Annexin V-PI apoptosis assay kits were used to assess apoptotic induction in control, vector, and F11R knockdown cells by flow cytometry (Figures 5(a) and 5(b)). After repeated three experiments, the results showed that the apoptosis rate of PANC-1 cells in the F11R-KD group was 11.97 ± 0.56%, while the apoptosis rate of PANC-1 cells in the vector group and control group was 1.49% ± 0.32% and 1.93% ± 0.64%, respectively. It can be seen that after F11R gene was knock down, the apoptosis rate of cells increased significantly, and the difference was statistically significant (*P* < 0.01).

### 3.6. Knockdown of F11R Inhibited PANC-1 Cell Migration

**Figure 6:**
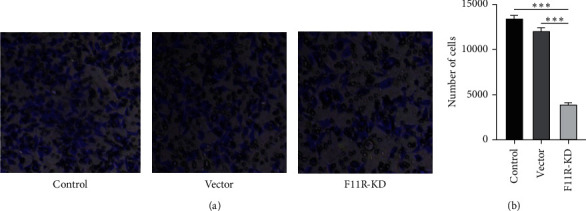
PANC-1 cell migration in vitro. In the F11R-KD group, the number of cells was significantly decreased by transwell assay, indicating a loss of invasion. Cell numbers were calculated using ImageJ. Transwell assays showed that the number of migrating cells significantly decreased following F11R knockdown (^*∗∗∗*^*P* value <0.001).

As metastasis is a key event in tumor progression and is responsible for most deaths in cancer patients, the migrating ability of PANC-1 cells with F11R gene knockdown was explored. This experiment was performed using the transwell method to detect the migration ability of PANC-1 cells. After 24 hours of incubation in a transwell chamber, the transfected cells were stained and counted. As shown in Figures 6(a) and 6(b), the results showed that, compared with the control group and vector group, the number of metastatic cells in the F11R-KD group was significantly decreased, and the difference was statistically significant (*P* < 0.01). The results indicated that the knockdown of the F11R gene can inhibit the migration of PANC-1 cells in vitro.

## 4. Discussion

Pancreatic cancer is projected to surpass breast, prostate, and colorectal cancers to become the second leading cause of cancer-related deaths behind lung cancer by 2030, even though breast, prostate, and lung cancers will remain the top cancer diagnoses throughout this time [23]. Although significant progress has been made in the development of novel cancer therapies, pancreatic cancer survival rates have failed to improve in the last 40 years [24]. Current treatments for pancreatic cancer include surgery, radiotherapy, chemotherapy, and gene therapy. Compared to the other therapy methods, gene therapy has many advantages, including low cytotoxicity, low side effects, and high tolerance. The F11R receptor belongs to the immunoglobulin superfamily and is expressed in epithelial and endothelial cells [25]. F11R mediates the formation of tight junctions between the epithelium and endothelium and participates in the invasion and metastasis of tumor cells [10]. F11R localizes to microtubules and plays an important role in cell-to-cell adhesion [26]. There are already reports that F11R antagonists are therapeutic agents for the prevention and treatment of thrombosis, atherosclerosis, heart attacks, strokes, and other clinical disorders [27–30], but whether F11R antagonists can be used in treating malignant tumors is still prelimited. However, some research groups have tried to study the role of F11R gene expression in tumor progression. Goetsch et al. [31] found that F11R monoclonal antibodies could reduce MCF-7 breast cancer cell proliferation. Phosphoinositide 3-kinase (PI3K) and protein kinase C (PKC) inhibitors could prevent F11R-induced migration that provided mechanistic insight into its oncogenic effects. However, the behavioral changes in pancreatic cancer cells caused by the expression of F11R remain unclear, and the identification of F11R as a novel antipancreatic cancer target therefore holds interest. This will be the focus of our future pancreatic cancer studies.

Through pre-experiment, we found that the F11R gene was highly expressed in five pancreatic cancer cell lines (MIA paca-2, bxpc-3, cfpac-1, SW1990, and PANC-1) and tumor specimens from 30 pancreatic cancer patients (data not shown). All of our experimental results can also be seen in the preprint (v1_covered.pdf (https://researchsquare.com) [32]. As one of the most commonly used cell models, and considering the expression level of F11R, PANC-1 was used as the model in all our experiments. Our results showed that the F11R-shRNA lentivirus effectively knocks down the mRNA and protein expression of the F11R gene by qPCR and western blot assays. Then, it was found that the knockdown of F11R revealed that the growth of the pancreatic cancer cell line PANC-1 was inhibited by CCK-8 assays. In order to explore the reasons for the knockdown of the F11R gene to induce cell growth inhibition, the cells were further assayed for apoptosis, cell cycle, and cell clone formation. In this study, apoptosis-related experiments found that knockdown of the F11R gene effectively activated apoptosis. The results of cell cycle experiments showed that the number of cells in the G1 phase increased, the number of cells in the S phase decreased, and the number of cells in the G2/M phase was similar. The results indicated that the knockdown of the F11R gene arrested the pancreatic cancer cell cycle in the G1 phase. In addition, the results of colony formation experiments showed that after the knockdown of the F11R gene, the viability of individual cells decreased significantly, and the ability to form clones in the experiment group was significantly inhibited. The above experiments showed that F11R knockdown may increase apoptosis by activating the apoptotic system and inhibit the cell cloning ability, thereby inhibiting cell proliferation. A number of studies have proved that the enhancement of intracellular reactive oxygen species (ROS) was one of the important characteristics of cell apoptosis [30], and oxidative stress caused lipid peroxidation, protein denaturation, and DNA damage of cell [29], which leads to apoptosis [30, 31]. Our results confirm that a large amount of ROS is produced in cells after the knockdown of F11R [1]. However, the relationship between decreased F11R expression and increased ROS remains to be further studied.

Metastasis is one of the characteristics of malignant behavior in pancreatic cancer [34]. In order to investigate whether the F11R gene affects the metastatic ability of pancreatic cancer cells, the transwell assay was carried out. The results showed that the migration ability of pancreatic cancer cells in the F11R-KD group knocking down the F11R gene was significantly decreased, indicating that silencing the F11R gene can effectively inhibit the metastatic ability of pancreatic cancer cells.

In conclusion, the results of this study showed that the knockdown of the F11R gene can be effectively silenced by lentiviral transfection of the pancreatic cancer cell line PANC-1 in vitro. The knockdown of F11R can inhibit cell proliferation and colony formation, promote apoptosis, and inhibit metastasis in pancreatic cancer cell lines PANC-1. Therefore, the knockdown of F11R gene-targeted therapy can be a possible therapeutic approach in patients with high expression of the F11R gene. Further experimental investigation into how the F11R gene influences the molecular pathway of the PANC-1 cell line signaling pathway is needed.

## Data Availability

UALCAN analysis: the expression levels of F11R and tumor grade were analyzed in UALCAN database (https://ualcan.path.uab.edu/) [18]. GEPIA (Gene Expression Profiling Interactive Analysis) (https://gepia.cancer-pku.cn/) [19] is an interactive web application for gene expression analysis based on 9736 tumors and 8587 normal samples from the TCGA (The Cancer Genome Atlas) and the GTEx (Genotype-Tissue Expression) databases. The GEPIA database was used to compare mRNA levels of F11R between tumor and normal tissue in TCGA databases. Kaplan–Meier plotter (KM plotter; https://kmplot.com/analysis/) [20] databases were leveraged to quantify associations between F11R expression and PC patient prognosis.
